# Impact of waist-to-hip and waist-to-height ratios on physical performance: insights from the Longevity Check-up 8+ project

**DOI:** 10.18632/aging.206260

**Published:** 2025-05-30

**Authors:** Anna Maria Martone, Elena Levati, Francesca Ciciariello, Vincenzo Galluzzo, Sara Salini, Riccardo Calvani, Emanuele Marzetti, Francesco Landi

**Affiliations:** 1 Fondazione Policlinico Universitario “Agostino Gemelli” IRCCS, Rome 00168, Italy; 2 Department of Geriatrics, Orthopedics and Rheumatology, Università Cattolica del Sacro Cuore, Rome 00168, Italy

**Keywords:** physical performance, body composition, waist-to-hip ratio, waist-to-height ratio, chair-stand test

## Abstract

Background: Physical performance is crucial for healthy aging. Body composition has gained particular attention. Anthropometric measurements, specifically the waist-to-hip ratio (WHR) and the waist-to-height ratio (WHtR), have emerged as valuable indicators. This study aims to investigate the correlation between abnormal WHR and waist-to-height ratios with physical performance.

Methods: Data from the Longevity Check-up 8+ project were analyzed. Anthropometric measurements were used to calculate WHR and WHtR. Physical performance was evaluated through the chair stand test. ANCOVA assessed the impact of WHR and WHtR on physical performance, while Cox proportional-hazards models were used to assess the relation between WHR, WHtR and physical performance. ROC curves analyzed their predictive capability.

Results: Among 10690 participants (mean age 57.0 ± 14.8 y; 54% females), men exhibited higher WHR and WHtR and a higher prevalence of abnormal values (61% and 71%). Women took longer to complete the chair stand test (7.9 ± 2.7 vs. 7.6 ± 2.4 seconds, *p* < 0.01). Abnormal WHR and WHtR were associated with poorer physical performance after adjusting for confounders (HR: 1.28; 95% CI: 1.08–1.53; HR: 1.32; 95% CI: 1.04–1.66). ROC curve analysis showed that WHtR had superior predictive capability to identify lower physical performance across age and gender groups.

Conclusions: Individuals with higher WHR and WHtR values demonstrated poorer physical performance, underscoring the importance of monitoring abdominal fat distribution as a predictor of functional health and aging-related outcomes.

## INTRODUCTION

Physical performance, encompassing various aspects of fitness and functionality, plays a pivotal role in determining an individual’s overall health and well-being [1, 2]. As researchers and healthcare professionals strive to better understand the factors influencing physical performance, the relationship between body composition and performance outcomes has gained considerable attention. Within the realm of assessing body composition through anthropometric measurements, two prominent metrics have garnered increasing attention: the waist-to-hip ratio (WHR) and, more recently, the waist-to-height ratio (WHtR) [3]. These measurements have risen to prominence as potentially valuable indicators due to their capacity to provide insights into the distribution of body fat and overall health status. Their utilization holds significant promise in both research and clinical settings [4, 5], offering a comprehensive perspective on individuals’ body composition and its potential health risks [6, 7].

The waist-to-hip ratio, defined as the ratio of waist circumference to hip circumference, has been recognized as an indicator of central adiposity and fat distribution. Its significance extends beyond aesthetics, as an elevated WHR has been associated with an increased risk of various health conditions, including cardiovascular diseases, metabolic disorders, and impaired glucose tolerance [8, 9]. Similarly, the waist-to-height ratio (WHtR) is also crucial in clinical practice as it offers a direct and efficient assessment of an individual’s health risks associated with abdominal fat. This measurement provides insights into the risk of conditions like cardiovascular diseases and diabetes [10, 11], making it a valuable tool for healthcare professionals to gauge health status and potential risks quickly.

Recognizing the correlation between WHR, WHtR, and physical performance holds special significance within aging demographics [12]. As life expectancy rises worldwide, safeguarding physical function and autonomy among older individuals takes on pronounced public health importance. Shifts in body composition due to aging, characterized by heightened central adiposity, can markedly influence physical performance and functional autonomy. Furthermore, evaluating both WHR and WHtR in adult subjects is particularly pertinent when considering the goal of promoting health longevity. In an era of increasing life expectancy, focusing on these metrics takes on added significance as they provide valuable insights into an individual’s risk for age-related health issues.

The present study aims to improve comprehension of the occurrence of abnormal waist-to-hip (WHR) and waist-to-height (WHtR) ratios, as well as their correlation with physical performance, particularly the 5-repetition chair stand test. This goal will be achieved through an examination of data derived from an unbiased selection of individuals who underwent evaluation as part of the Longevity Check-up 8+ project.

## MATERIALS AND METHODS

The data employed in this study were derived from the Lookup 8+ initiative, which is endorsed by the Department of Geriatrics at Fondazione Policlinico “Agostino Gemelli” IRCCS, Università Cattolica del Sacro Cuore in Rome, Italy [13]. The primary objective of the Lookup 8+ project was to promote the adoption of healthy lifestyles and to raise awareness on cardiovascular risk factors within the general population. Participants were recruited from public places (such as exhibitions and shopping centers) and individuals engaging in prevention campaigns initiated by our institution. These locations were chosen because it allowed the enrolment of relatively unselected participants, outside of conventional healthcare or research settings.

The Lookup 8+ protocol received ethical approval from the Ethics Committee of Università Cattolica del Sacro Cuore (protocol #: A.1220/CE/2011) and is comprehensively detailed elsewhere [13, 14]. This manuscript adheres to the STrengthening the Reporting of OBservational studies in Epidemiology (STROBE) reporting guidelines for observational studies [15].

### Study sample

From January 1st, 2018, to August 31st, 2024, a total of 10,934 individuals were recruited from various Italian cities as part of the national Lookup 8+ project. To be eligible for participation in the Lookup 8+ screening, individuals had to be at least 18 years old and provide written informed consent. Exclusion criteria encompassed self-reported pregnancy, inability to execute the physical performance test (e.g., wheelchair-bound), or incapacity to provide written informed consent.

In the present study, our specific focus was on individuals who underwent a thorough anthropometric evaluation, encompassing measurements of both waist and hip circumferences. After excluding participants with any missing data in the variable of interest, the final sample comprised 10690 participants.

### Data collection

Individuals who willingly participated in the screening process underwent comprehensive assessments, which included a concise questionnaire, the measurement of objective cardiovascular health indicators, and evaluations of anthropometric measures such as height, weight, waist, hip and lower extremity muscle power. The Lookup 8+ visit was organized to gather an array of vital information and data, encompassing informed consent, a lifestyle interview (covering smoking, dietary habits, physical activity), blood pressure assessment, weight and height measurements (used to calculate BMI), waist and hip circumferences and assessments of cholesterol and glucose levels [16, 17].

Regarding specific details, smoking status was categorized as either a current smoker or never/former smoker. Body weight was measured using an analog medical scale, while body height was assessed with a standard stadiometer. Body mass index (BMI) was calculated by dividing weight (in kilograms) by the square of height (in meters). A healthy diet was defined as consuming a minimum of three servings of fruits and/or vegetables per day [18]. Regular participation in physical activity was defined as engaging in exercise training at least twice weekly. Cholesterol levels were determined using capillary blood samples and a reflectometric system employing portable device MultiCare-In [19] and Wellion Galileo GLU/KET [20]. Similarly, random blood glucose levels were measured from capillary blood samples using changing reagent strips and an amperometric system with the portable device MultiCare-In [19] and Wellion Galileo GLU/KET [20]. Blood pressure measurements adhered to international guidelines and were taken with a manual sphygmomanometer [21].

### Waist-to-hip and waist-to-height ratios assessment

The waist circumference was measured at the narrowest point between the ribs and hips [22]. The individual stood with feet together and exhaled gently. The measuring tape was comfortably snug but it didn’t press into the skin. The hip circumference was measured at the widest part of the buttocks [22]. The measuring tape was positioned parallel to the ground and encircled the hip bones. The individual’s height was taken using a standard stadiometer with the individual stand upright without shoes.

The WHR was calculated dividing the waist measurement by the hip measurement (WHR = Waist Circumference/Hip Circumference). The WHtR was calculated dividing the waist measurement by the height measurement (WHtR = Waist Circumference/Height).

For men, a WHR below 0.90, and for women, a WHR below 0.85, has been considered normal [3]. Additionally, a WHtR below 0.5, applicable to both men and women, has been recognized as a normal value, indicative of a more favorable distribution of body fat [23].

### Physical performance assessment

During the Lookup 8+ screening, participants’ physical abilities were assessed through the chair stand test. In this test, individuals were instructed to rise from an armless chair with their arms folded across their chest, completing the motion five times consecutively as swiftly as possible. Standardized instructions were provided to ensure consistency across participants. Attention was paid to ensure the correct posture during the test, but slight variations in technique were allowed to accommodate individual differences. A standard chair of approximately 43–47 cm in height was utilized. To ensure safety and stability, the back of the chair was secured against a wall. A handheld stopwatch was employed to measure the time taken to complete the task. Widely recognized for its reliability and validity, the five-repetition chair stand test serves as a straightforward measure of physical function among adults and older individuals, even encompassing those with musculoskeletal or neurological conditions [12, 24]. A completion time surpassing 10.8 seconds has been identified as a marker of suboptimal physical performance, signifying potential mobility challenges and subsequent disability [25].

### Statistical analysis

The study participants’ characteristics were described by gender. Descriptive statistics were computed for the data. The Kolmogorov-Smirnov test was utilized to verify the normal distribution of continuous variables. Mean values with standard deviations were presented for continuous variables, while categorical variables were shown as absolute numbers along with percentages. Fisher’s exact test assessed differences in categorical variables, while the one-way analysis of variance (ANOVA) or Kruskal-Wallis test was applied for continuous variables. In all cases, statistical significance was defined at *p* < 0.05.

Waist-to-hip ratio (WHR) and waist-to-height ratio (WHtR) were established as independent variables, with physical performance (chair stand test) serving as the dependent variable. Analysis of covariance (ANCOVA) was executed to explore the relationship between various anthropometric assessments (WHR and WHtR) and physical performance. The assumption of normality was validated by reviewing histograms of the dependent variable and residuals. Variables with potential correlations to physical performance (smoking habit, physical activity, healthy diet, BMI, systolic and diastolic blood pressure, cholesterol, and glucose levels) were considered for adjustment.

Using Cox proportional-hazards models, we computed both unadjusted and adjusted hazard ratios (HRs) along with their corresponding 95% confidence intervals (CIs) to assess the impact of WHR and WHtR levels on poor physical performance. Variables possibly linked to diminished physical performance were incorporated into the models. The ultimate analyses were thus fine-tuned through successive adjustments: initially for age and gender (Model 1); subsequently for age, gender, smoking habit, healthy diet, and physical activity (Model 2); and finally, for age, gender, smoking habit, healthy diet, physical activity, BMI, blood pressure, cholesterol, and glucose levels (Model 3).

Finally, in order to evaluate the predictive capability of WHR and WHtR (independent variables) for indicating poor physical performance (dependent variable), receiver operating characteristic (ROC) curves were constructed. The resulting area under the curve (AUC) was reported, and the sensitivity and specificity at the respective WHR and WHtR thresholds were determined.

All statistical analyses were conducted using SPSS software (version 11.0, SPSS Inc., Chicago, IL, USA).

## RESULTS

The study encompassed a total of 10690 participants, with a mean age of 57.0 ± 14.8 years (range: 18–98). Among them, 5820 (54%) were women. The principal characteristics of the study population are detailed based on gender in Table 1. In comparison to females, males exhibited higher engagement in physical activity, and were less likely to follow a healthy diet. Men also demonstrated higher BMI, systolic and diastolic blood pressure, as well as serum glucose levels. Conversely, serum cholesterol levels were higher among female participants. Notably, the two anthropometric measures under consideration, WHR and WHtR, were significantly greater in men than in women. Interestingly, abnormal values for WHR and WHtR were significantly more prevalent among men, accounting for 61% and 71%, respectively. The time taken to complete the five-repetition chair stand test was longer among women compared to men (7.9 ± 2.7 seconds vs. 7.6 ± 2.4 seconds, *p* < 0.01). In terms of mean age and the prevalence of smoking habits, no substantial differences were observed between the two groups of interest.

**Table 1:** Characteristics of study population according to gender^*^.

**Characteristics**	**Total sample (*n* = 10690)**	**Male (*n* = 4870)**	**Female (*n* = 5820)**	***p***
**Age (years)**	57.0 ± 14.8	57.3 ± 15.0	56.7 ± 14.8	0.07
<65 years	7271 (67)	3254 (67)	3997 (69)	
>65 years	3419 (33)	1616 (33)	1823 (31)	
**Smoking**	2104 (20)	965 (20)	1139 (20)	0.39
**Healthy diet**	6626 (62)	2682 (55)	3944 (68)	<0.001
**Physically active**	5904 (56)	3041 (63)	2863 (50)	<0.001
**BMI (Kg/m^2^)**	24.8 ± 3.9	25.6 ± 3.5	24.0 ± 4.2	<0.001
**Cholesterol (mg/dl)**	192.6 ± 36.7	184.7 ± 36.1	199.2 ± 35.9	<0.001
**Serum glucose (mg/dl)**	106.0 ± 23.5	108.1 ± 25.8	104.2 ± 21.2	<0.001
**Systolic blood pressure (mmHg)**	124.7 ± 16.3	128.4 ± 15.7	121.6 ± 16.1	<0.001
**Diastolic blood pressure (mmHg)**	75.8 ± 9.9	77.7 ± 9.7	74.2 ± 9.8	<0.001
**WHR**	0.87 ± 0.09	0.92 ± 0.07	0.83 ± 0.08	<0.001
**Abnormal WHR**	5207 (49)	2978 (61)	2229 (39)	<0.001
**WHtR**	0.52 ± 0.07	0.54 ± 0.07	0.51 ± 0.08	<0.001
**Abnormal WHtR**	6560 (61)	3481 (71)	3079 (53)	<0.001
**Chair Stand test (sec)**	7.8 ± 2.6	7.6 ± 2.4	7.9 ± 2.7	<0.01

^*^Data are given as number (percent) for age group, gender, smoking, healthy diet, physical activity, abnormal WHR and abnormal WHtR; for all the other variables, means ± SD are reported. Abbreviations: Healthy diet: consumption of at least three portions of fruit and/or vegetables per day; Physically active: physical exercise at least twice a week; BMI: Body mass index; WHR: Waist-to-hip ratio; Abnormal WHR: above 0.85 in women and 0.90 in men; WHtR: Waist-to-height ratio; Abnormal WHtR: above 0.5.

**Figure 1:**
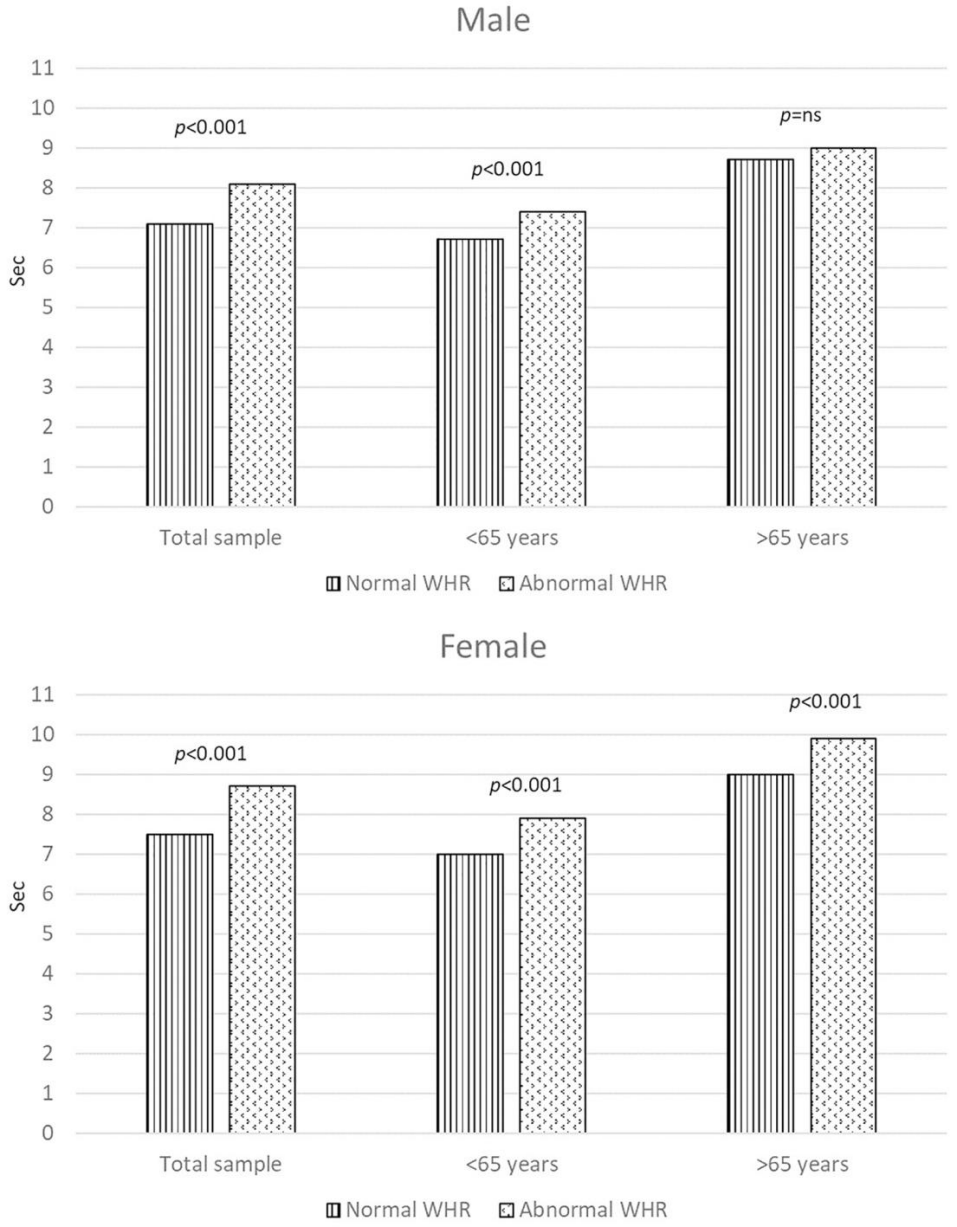
**The figure illustrates the assessment of physical performance using the chair stand test (in seconds) categorized by normal and abnormal waist-to-hip ratios within the entire study population and across distinct age groups.** Panel A pertains to male participants, while Panel B pertains to female participants. The analysis of covariance (ANCOVA) was adjusted for smoking habit, physical activity, healthy diet, BMI, systolic and diastolic blood pressure, cholesterol, and glucose levels.

**Figure 2:**
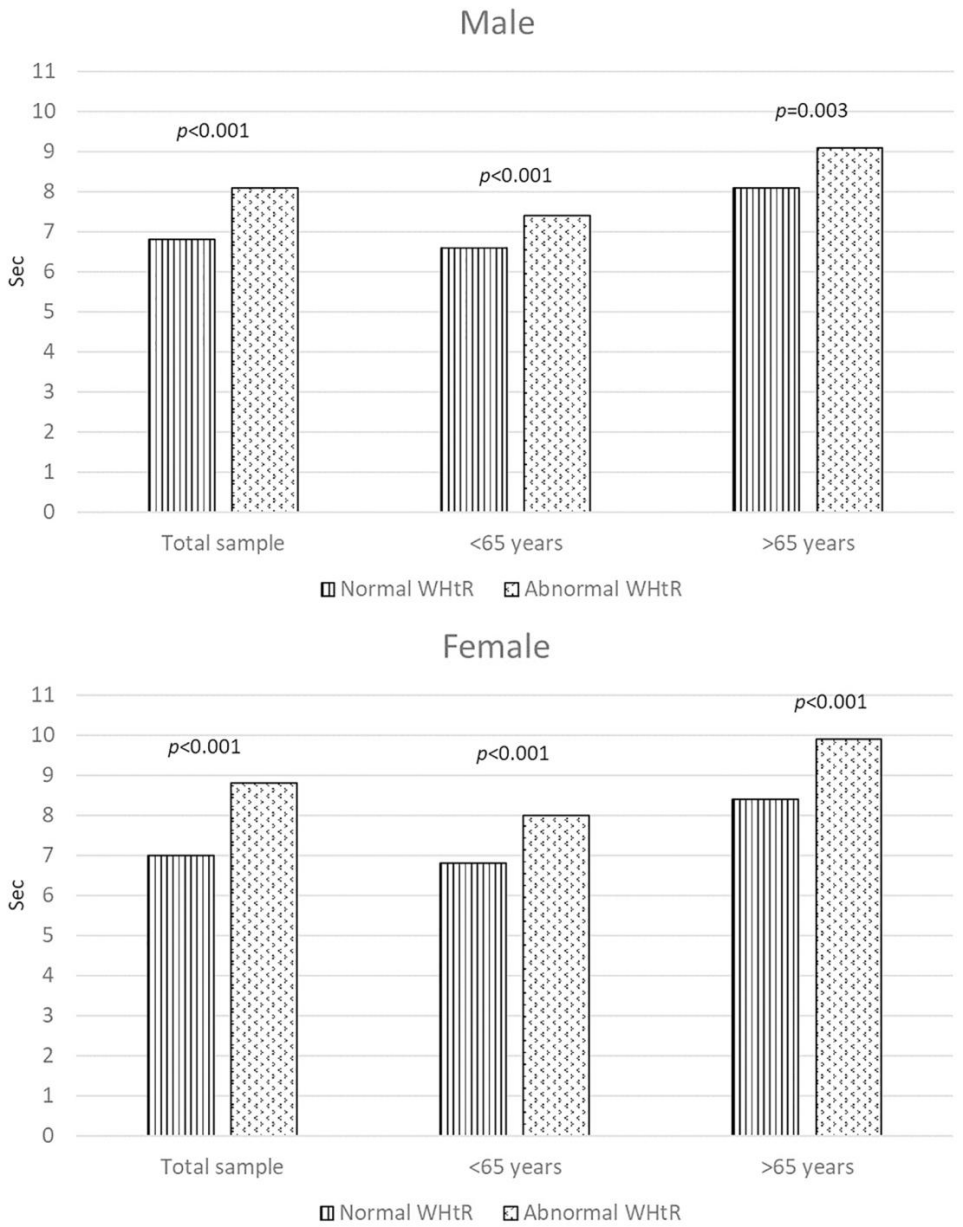
**The figure illustrates the assessment of physical performance using the chair stand test (in seconds) categorized by normal and abnormal waist-to-height ratios within the entire study population and across distinct age groups.** Panel A pertains to male participants, while Panel B pertains to female participants. The analysis of covariance (ANCOVA) was adjusted for smoking habit, physical activity, healthy diet, BMI, systolic and diastolic blood pressure, cholesterol, and glucose levels.

Figure 1 displays the adjusted outcomes derived from ANCOVA models, revealing variations in physical performance between individuals with abnormal and normal WHR. Following adjustments for smoking habit, physical activity, healthy diet, BMI, systolic and diastolic blood pressure, cholesterol, and glucose levels, noteworthy differences in the duration taken to complete the five-repetition chair stand test across different WHR categories were evident. This trend remained consistent among both male and female participants, excluding men aged 65 years and older, where individuals with an abnormal WHR exhibited poorer performance. Figure 2 shows the variations in physical performance between individuals with abnormal and normal WHtR. Following adjustments for the same variables, distinct distributions in the duration taken to complete the five-repetition chair stand test across different WHtR categories were consistent across both male and female participants, regardless of age group.

The outcomes from both unadjusted and adjusted Cox proportional hazard models are presented in Table 2. In the unadjusted analysis, a clear positive link was observed between abnormal WHR and WHtR and poor physical performance (HR: 2.48; 95% CI: 2.16–2.84 and HR: 4.27; 95% CI: 3.57–5.10, respectively). Even after accounting for potential confounding factors (age, gender, smoking habit, healthy diet, physical activity, BMI, blood pressure, cholesterol, and glucose levels), this association retained its statistical significance (Table 2). In the fully adjusted model, participants exhibiting abnormal WHR and WHtR displayed an elevated risk of lower physical performance in comparison to those with normal WHR and WHtR (HR: 1.28; 95% CI: 1.08–1.53; HR: 1.32; 95% CI: 1.04–1.66, respectively).

**Table 2:** Crude and adjusted hazard ratio of poor physical performance and 95% confidence intervals in the study population.

	**Unadjusted**	**Model 1**	**Model 2**	**Model 3**
	***Hazard ratio* (*95% confidence interval*)**			
**Abnormal WHR**	**2.48 (2.16–2.84)**	**1.62 (1.38–1.87)**	**1.58 (1.35–1.85)**	**1.28 (1.08–1.53)**
Age		1.08 (1.07–1.09)	1.08 (1.07–1.09)	1.08 (1.07–1.09)
Gender (female)		0.58 (0.50–0.67)	0.63 (0.55–0.73)	0.60 (0.51–0.71)
Smoking habit			1.07 (0.89–1.29)	1.09(0.89–1.32)
Healthy diet			0.85 (0.73–0.99)	0.85 (0.72–0.99)
Physical activity			0.52 (0.45–0.60)	0.59 (0.50–0.69)
BMI (Kg/m^2^)				1.07 (1.05–1.09)
Systolic blood pressure (mmHg)				1.00 (0.99–1.01)
Diastolic blood pressure (mmHg)				0.99 (0.98–1.01)
Cholesterol (mg/dl)				1.00 (0.99–1.01)
Glucose (mg/dl)				1.00 (0.99–1.01)
**Abnormal WHtR**	**4.27 (3.57–5.10)**	**2.25 (1.85–2.72)**	**2.07 (1.69–2.52)**	**1.32 (1.04–1.66)**
Age		1.08 (1.07–1.09)	1.08 (1.07–1.09)	1.08 (1.07–1.09)
Gender (female)		0.58 (0.50–0.67)	0.63 (0.54–0.73)	0.61 (0.52–0.72)
Smoking habit			1.09 (0.90–1.31)	1.10 (0.90–1.33)
Healthy diet			0.86 (0.74–0.99)	0.84 (0.72–0.98)
Physical activity			0.53 (0.48–0.64)	0.60 (0.51–0.70)
BMI (Kg/m^2^)				1.07 (1.05–1.09)
Systolic blood pressure (mmHg)				1.00 (0.99–1.01)
Diastolic blood pressure (mmHg)				0.99 (0.98–1.01)
Cholesterol (mg/dl)				1.00 (0.99–1.01)
Glucose (mg/dl)				1.00 (0.99–1.01)

Abbreviations: WHR: Waist-to-Hip ratio; WHtR: Waist-to-Height ratio.

**Figure 3:**
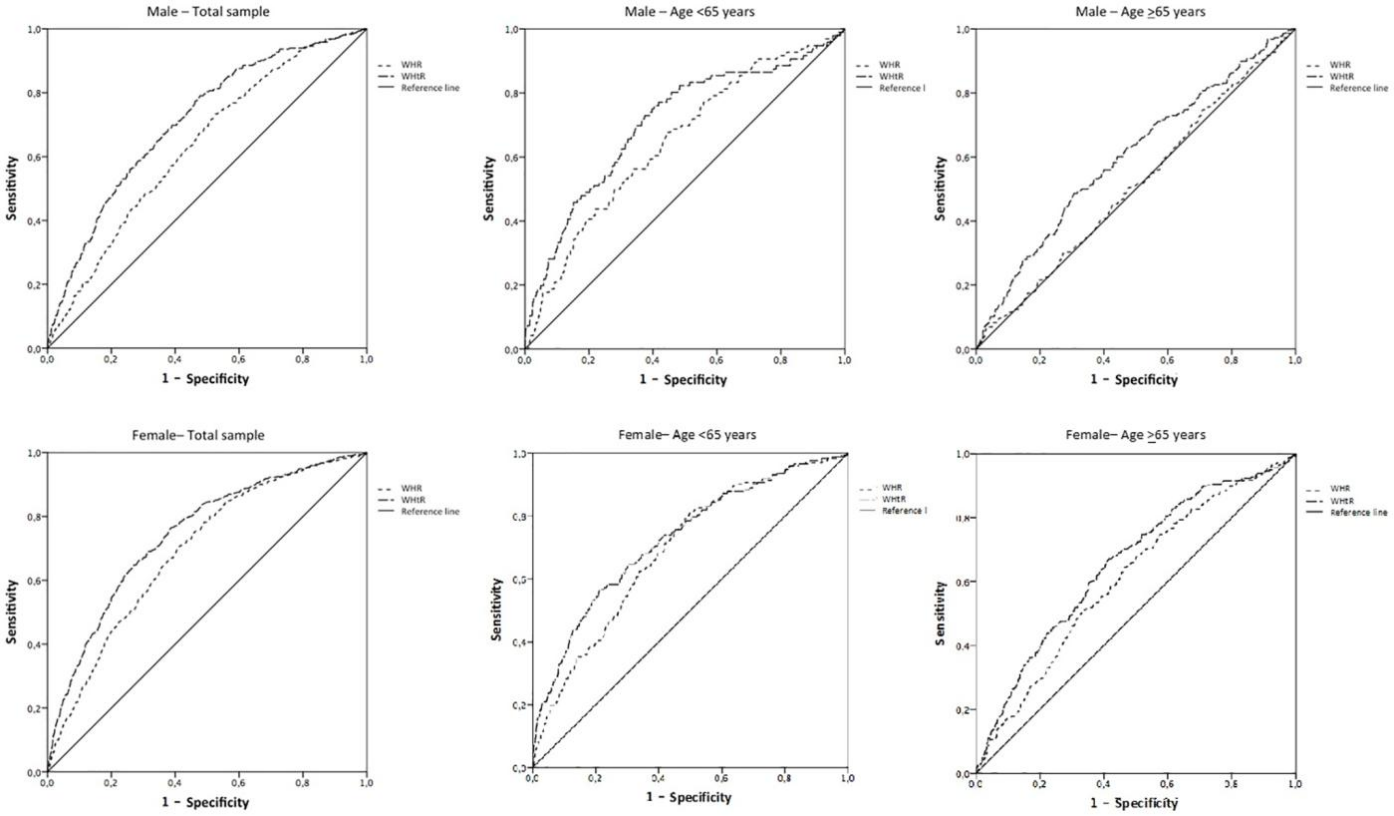
**Receiver operating characteristic (ROC) curve analysis for predicting lower physical performance (by means time to complete chair stand test more than 10.8 seconds) according to waist-to-hip (WHR) and waist-to-height (WHtR) ratios.** The ROC curve analysis revealed that the areas under the curves (AUCs) were: (1) Male total sample: WHR = 0.63; WHtR = 0.70. (2) Male age <65 years: WHR = 0.64; WHtR = 0.70. (3) Male age >65 years: WHR = 0.51; WHtR = 0.60. (4) Female total sample: WHR = 0.69; WHtR = 0.75. (5) Male age <65 years: WHR = 0.69; WHtR = 0.73. (6) Male age >65 years: WHR = 0.60; WHtR = 0.67.

Finally, receiver operating characteristic (ROC) curve analysis was conducted to predict lower physical performance (defined as taking more than 10.8 seconds to complete the chair stand test) based on waist-to-hip ratio (WHR) and waist-to-height ratio (WHtR) (Figure 3). The results of the ROC curve analysis consistently demonstrated that the areas under the curves (AUCs) were consistently greater for WHtR in comparison to WHR. This trend held true across gender and age groups, indicating the superior predictive ability of WHtR for identifying lower physical performance. Considering the WHR cut-off value of 0.90 in male, the sensitivity and specificity were 82% and 64%, respectively. Similarly, considering the cut-off value of 0.85 in female, the sensitivity and specificity were 67% and 39%, respectively. On the other hand, considering the WHtR threshold of 0.50 for both man and women the sensitivity and specificity were 87% and 64%.

## DISCUSSION

The present study aimed to investigate the association between waist-to-hip ratio (WHR), waist-to-height ratio (WHtR) and physical performance in individuals of varying age groups. The findings of this research contribute to the growing body of evidence regarding the relationship between body composition and physical capabilities.

Our results demonstrated a significant correlation between these anthropometric measures and physical performance across the study population. Specifically, individuals with higher WHR and WHtR values exhibited poorer physical performance compared to those with lower WHR and WHtR values, respectively. This association was observed across different gender and age groups, indicating that these simple measures may have an impact on physical performance.

One possible explanation for the observed association is the distribution of adipose tissue in the abdominal region. A higher WHR and/or WHtR typically indicates a higher amount of abdominal fat, which has been associated with various health risks and decreased physical function [12]. Excess abdominal fat is known to contribute to decreased muscle strength, flexibility, and overall mobility, which can subsequently hinder physical performance [26, 27]. Moreover, the accumulation of visceral fat around vital organs may impair cardiovascular function and metabolic health, leading to decreased endurance and aerobic capacity. This can further contribute to reduced physical performance in tasks requiring sustained effort, such as long-distance running or prolonged exercise.

It is worth noting that while our study establishes a correlation between WHR and WHtR and physical performance, the underlying mechanisms responsible for this association require further investigation. Future research should explore potential mediators, such as muscle mass, muscle quality, and inflammation markers, to gain a better understanding of the physiological pathways linking abdominal adiposity and physical performance.

In fact, abdominal fat could be linked with fat infiltration in muscle. Muscle quality is precisely impaired in sarcopenia which is defined as “a progressive and generalized skeletal muscle disorder that is associated with increased likelihood of adverse outcomes including falls, fractures, physical disability and mortality (EGSWOP2)”. Therefore, abdominal fat could correlate with fat infiltration of muscle which is one of the major characteristics of sarcopenia. Sarcopenia in turn is strongly associated with physical performance and health outcomes (EGSWOP2).

The findings of this study have practical implications for individuals aiming to improve their physical performance and overall health. By addressing abdominal obesity and reducing waist circumference through appropriate lifestyle interventions, such as regular exercise and a balanced diet, individuals may enhance their physical capabilities and reduce the risk of age-related functional decline. In particular, in the realm of predicting physical performance, WHtR emerges as a notably superior metric when compared to WHR. The WHtR offers distinct advantages in terms of both its predictive accuracy and its practical applicability within clinical settings. Our results consistently demonstrate that WHtR exhibits enhanced discriminatory power, exemplified by consistently higher area under the curve (AUC) values in receiver operating characteristic (ROC) analyses. This impressive predictive capacity highlights how WHtR efficiently categorizes individuals based on their physical performance levels. Furthermore, the straightforward measurement process of WHtR enhances its practical value in clinical settings. Calculating WHtR requires only waist and height measurements, promoting its implementation and eliminating the complexities associated with measuring WHR. Consequently, WHtR not only surpasses WHR in predictive accuracy but also provides a simplified and practical method for assessing potential mobility issues. This makes it a valuable tool for clinicians and researchers aiming to evaluate and forecast physical performance across various populations.

This takes on strong relevance from a predictive point of view if we consider that physical performance is associated with health outcomes (EGSWOP2).

It is important to acknowledge the limitations of our study. Firstly, the cross-sectional design restricts the establishment of causality between WHR and WHtR with lower physical performance. Additionally, the study population included almost exclusively Caucasians and the results may not be generalizable to other racial groups. The requirement for participants to reach the recruitment site likely selected for a relatively healthy and functionally competent population. Although the chair stand test was conducted by trained investigators using a standardized protocol, activities performed prior to the assessment (e.g., carrying bags, walking, resting) may have an impact on their performance levels. Furthermore, despite following standardized guidelines for measuring height, waist and hip circumferences, the study setting may have been influenced the measurement outcomes.

In conclusion, our study provides evidence of a significant association between WHR and WHtR with physical performance, suggesting that abdominal obesity may adversely impact an individual’s physical capabilities. These findings emphasize the importance of maintaining a healthy body composition and waist circumference for optimal physical function and the value of WHR and WHTR in predicting health outcomes. Further research is warranted to elucidate the underlying mechanisms and explore potential interventions to mitigate the negative effects of abdominal obesity on physical performance. Longitudinal studies to analyze the potential causality between WHR/WHtR and physical performance should be considered. Ultimately, this research can help inform clinical practice and public health initiatives aimed at promoting healthy aging and optimizing physical performance outcomes. By assessing WHR and WHtR, healthcare practitioners can identify individuals at greater risk of these health complications and implement targeted interventions to mitigate risks and improve long-term health prospects. These measurements serve as easily accessible tools that empower individuals to make informed lifestyle choices, contributing to their overall well-being and the potential for a healthier and longer life.
